# Temporal variation, socioeconomic status, and out‐of‐hospital deaths as factors that influence mortality rates among hospitalized COVID‐19 patients receiving ACEIs/ARBs

**DOI:** 10.1111/jch.14473

**Published:** 2022-03-21

**Authors:** Owais M. Aftab, Anurag Modak, Jai C. Patel

**Affiliations:** ^1^ Department of Medicine New Jersey Medical School Rutgers University Newark New Jersey USA; ^2^ Center for Advanced Biotechnology and Medicine Robert Wood Johnson Medical School Rutgers University Piscataway New Jersey USA

To the Editor,

We read with great pleasure the article by Jia, et al., titled “Influence of angiotensin converting enzyme inhibitors/angiotensin receptor blockers on the risk of all‐cause mortality and other clinical outcomes in patients with confirmed COVID‐19: A systemic review and meta‐analysis,” which found that angiotensin converting enzyme inhibitors (ACEIs) and angiotensin receptor blockers (ARBs) were not associated with in‐hospital mortality in COVID‐19 patients from March to November 2020.^1^ In fact, it was found that there may even be a decreased risk of all‐cause mortality in patients taking ACEIs or ARBs.^1^ Upon comparison with related trials and research on COVID‐19 in‐hospital mortality associated with ACEIs/ARBs, we would like to note the following limitations in interpreting the conclusions:

1. There is temporal variation in mortality that may complicate any conclusions made. The authors conducted an electronic search to include articles from the inception of the pandemic to November 12, 2020. However, mortality rates in the early stages of the pandemic in March 2020 differed significantly from mortality rates later on in October and November 2020.^2^ In fact, in‐hospital mortality rates decreased in the US over the course of 2020 from March to November, even after adjusting for age, sex, comorbidites, and disease severity at admission.^2^ As such, a retrospective analysis during this time period would be strengthened by accounting for this variation.
2. In addition, socioeconomic and sociocultural variations in mortality rates exist across countries. The hygiene hypothesis featured prominently during the early phase of the pandemic. Data from India's first COVID‐19 surge in October 2020 noted that infected individuals were protected from severe manifestations of the disease and experienced reduced mortality rates compared to Western nations due to cross‐immunity from other pathogens, which was confirmed serologically.^3^ More generally, it has been found that less‐developed nations with a higher burden of pediatric mortality as well as mortality from preventable disease had lower rates of COVID‐19 mortality.^4^ Furthermore, cultural differences between countries may play a role in the severity of the pandemic's impact; even prior to the COVID‐19 pandemic, countries with individualistic cultures may be at greater risk for an increase in outbreaks and transmission of infectious diseases when compared to collectivist cultures.^5^
3. The authors did not control for continuation or discontinuation of the ACEIs/ARBs after discharge from the hospital. A French study established a causal link between out‐of‐hospital cardiac arrest or out‐of‐hospital sudden death and COVID‐19 in the early pandemic, during March to April 2020 and some Indian studies found an increase in mortality associated with the administration of ACEIs/ARBs in hospitalized COVID‐19 patients in the early phase of the pandemic.^6^, ^7^, ^8^ However, a randomized controlled trial in Brazil found a non‐significant difference in mortality rates between the 30‐day continuation and 30‐day discontinuation groups.^9^ Although the results of the Brazilian study concord with the results of Jia et al., the clinical trial did not account for the same comorbidities as the retrospective study, so the authors of the present study missed an opportunity to more rigorously establish evidence in favor of use routine use of ACEIs/ARBs in hospitalized COVID‐19 patients with hypertension.^9^

Despite these limitations, the findings of the systematic review discussed does warrant further investigation, especially as the full breadth of COVID‐19‐related side‐effects is currently not understood. In developing nations, such as India, ACEIs/ARBs are not routinely given to hospitalized patients unless previously prescribed to the patients due to limitations on available medical resources,^7^, ^8^ so this area of research is particularly critical and the findings are immediately actionable.

## CONFLICTS OF INTERESTS

The authors declare that they have no conflicts of interests.
